# Breast Cancer Survival Defined by the ER/PR/HER2 Subtypes and a Surrogate Classification according to Tumor Grade and Immunohistochemical Biomarkers

**DOI:** 10.1155/2014/469251

**Published:** 2014-05-26

**Authors:** Carol A. Parise, Vincent Caggiano

**Affiliations:** Sutter Institute for Medical Research, 2801 Capitol Avenue, Suite 400, Sacramento, CA 95816, USA

## Abstract

*Introduction*. ER, PR, and HER2 are routinely available in breast cancer specimens. The purpose of this study is to contrast breast cancer-specific survival for the eight ER/PR/HER2 subtypes with survival of an immunohistochemical surrogate for the molecular subtype based on the ER/PR/HER2 subtypes and tumor grade. *Methods*. We identified 123,780 cases of stages 1–3 primary female invasive breast cancer from California Cancer Registry. The surrogate classification was derived using ER/PR/HER2 and tumor grade. Kaplan-Meier survival analysis and Cox proportional hazards modeling were used to assess differences in survival and risk of mortality for the ER/PR/HER2 subtypes and surrogate classification within each stage. *Results*. The luminal B/HER2− surrogate classification had a higher risk of mortality than the luminal B/HER2+ for all stages of disease. There was no difference in risk of mortality between the ER+/PR+/HER2− and ER+/PR+/HER2+ in stage 3. With one exception in stage 3, the ER-negative subtypes all had an increased risk of mortality when compared with the ER-positive subtypes. *Conclusions*. Assessment of survival using ER/PR/HER2 illustrates the heterogeneity of HER2+ subtypes. The surrogate classification provides clear separation in survival and adjusted mortality but underestimates the wide variability within the subtypes that make up the classification.

## 1. Introduction

It is generally acknowledged that breast cancer is a heterogeneous disease with a wide spectrum of clinical, pathologic, and molecular features [1–3]. The molecular classification is becoming the gold standard for complete characterization of breast cancer and the underlying technology has already generated gene-profiling models to predict outcomes [4–7]. Despite these remarkable achievements, in general, clinicians still rely on traditional clinicopathologic features and readily available tumor markers such as estrogen receptor (ER), progesterone receptor (PR), and human epidermal growth factor receptor 2 (HER2).

ER, PR, and HER2, routinely available in breast cancer specimens, are reliable, inexpensive, and useful for therapeutic decision making, and the results of these tests are recorded in cancer registries allowing for population-based research which make them a reasonable substitute for the more expensive molecular subtyping [8, 9]. There are eight combinations of ER, PR, and HER2 and significant differences in the demographics, tumor characteristics, and survival associated with these eight subtypes have been described for all stages combined and when stratified by St. Gallen risk categories [10, 11].

Gene expression profiling studies have identified at least four categories of breast cancer: luminal A, luminal B, HER2 overexpressing, and basal-like or triple negative (TN) [1, 2]. These molecular categories have been correlated with immunohistochemical (IHC) biomarkers [3, 9, 11–14]. However, the IHC correlate of luminal B remains imprecise. Some investigators consider any HER2+ tumor that is ER+ and/or PR+ to be luminal B [3, 15], but not everyone is in agreement [16]. Others have used Ki67 and HER2 to define two types of luminal B, one that is HER2− and with a high proliferation index as determined by Ki67, and one that is HER2+ [17, 18]. Tumor grade, instead of Ki67, and HER2 positivity may define similar luminal B phenotypes [19].

The purpose of this study is to contrast breast cancer-specific survival for the eight ER/PR/HER2 subtypes with survival of the IHC surrogates (surrogate classification) within American Joint Commission on Cancer (AJCC) stages 1, 2, and 3.

## 2. Methods

Using the population-based California Cancer Registry (CCR), we identified cases of primary first female invasive breast cancer (International Classification of Diseases for Oncology 3rd edition sites C50.0–C50.9) [20] diagnosed between January 1, 2000 and December 31, 2010 and reported to the CCR as of January, 2013. Cases are reported to the Cancer Surveillance Section of the California Department of Public Health from hospitals and any other facilities providing care or therapy to cancer patients residing in California [21]. Cases identified outside of California, only at autopsy, or only from death certificates were excluded. Breast cancer-specific mortality was defined as a death due to breast cancer as documented by the codes ranging from C50.01 to C50.91 of the International Statistical Classification of Diseases and Related Health Problems, 10th Revision [20]. Deaths due to causes other than breast cancer were censored.

### 2.1. Socioeconomic Status

Quintile of socioeconomic status (SES) was derived using data from the 2000 US census. SES was assigned at the census block group level and based on address at time of initial diagnosis, as reported in the medical record. This area based SES measure has been used in many studies utilizing cancer registry data [9, 13, 22–26]. A detailed description of this methodology is available [27].

### 2.2. Race/Ethnicity

Race/ethnicity was classified into five distinct categories: White, African American or black, Hispanic, Asian-Pacific Islander (API), and American Indian. A complete description of derivation of these categories is available in a previous publication [28].

### 2.3. ER/PR/HER2

The details of documentation of ER, PR, and HER2 along with age and stage at diagnosis and tumor grade have been extensively described in our previous publications and by the CCR [9–11, 13, 21, 29]. The eight subtypes were defined as ER+/PR+/HER2−, ER+/PR+/HER2+, ER+/PR−/HER2−, ER+/PR−/HER2+, ER−/PR+/HER2−, ER−/PR+/HER2+, ER−/PR−/HER2−, and ER−/PR−/HER2+.

### 2.4. Surrogate Classification

For this study, we defined the surrogate classification using the ER/PR/HER2 subtypes and tumor grade (low = tumor grade of 1 or 2; high = tumor grade of 3 or 4) [19]. Luminal A was classified as ER+/PR+/HER2−, ER+/PR−/HER2−, ER−/PR+/HER2−, and low tumor grade. Luminal B/HER2− was classified as ER+/PR+/HER2−, ER+/PR−/HER2−, and ER−/PR+/HER2− and has high tumor grade. Subtypes ER+/PR+/HER2+, ER+/PR−/HER2+, and ER−/PR+/HER2+ were classified as luminal B/HER2+. Triple negative was ER−/PR−/HER2−, and HER2 overexpressing was ER−/PR−/HER2+ (Table 1).

### 2.5. Statistical Analysis

Contingency tables were used to assess the distribution of demographic and tumor characteristics among the ER/PR/HER2 subtypes. Because of our interest in early breast cancer, only stages 1, 2, and 3 were included in bivariate and multivariate analyses.

Kaplan-Meier 5-year survival analysis and 95% confidence intervals (CI) and the Mantel-Cox Log Rank test were used to compare survival among categories of ER/PR/HER2 and surrogate classification for all stages combined and separately for stages 1, 2, and 3. A comparison was considered statistically significant if *P* < 0.05.

Cox proportional hazards modeling was used to determine time from breast cancer diagnosis to time of breast cancer death for each of the eight subtypes when compared with the ER+/PR+/HER2− subtype. Cox regression was also used to compare risk of mortality of the luminal B/HER2−, luminal B/HER2+, TN, and HER2 overexpressing classification when compared to luminal A. Analyses were conducted separately for stages 1, 2, and 3 because of the marked differences in prognosis of patients diagnosed in different stages. Analysis of the ER/PR/HER2 subtypes was adjusted for age, tumor grade, race/ethnicity, and SES. Analysis of the surrogate classification was similarly adjusted except for tumor grade since this was an intrinsic part of the luminal A and luminal B/HER2− categories. Hazard ratios (HR) and 95% CIs were computed.

This research study involved analysis of existing data from the CCR without subject identifiers or intervention. Therefore, the study was categorized as exempt from institutional review board oversight.

## 3. Results

There were 143,333 stages 1–4 cases with available ER, PR, and HER2 data (Table 2). Almost half of the cases were between the ages of 50 and 70 (48.8%). As age increased, the percent of women with the ER+/PR+/HER2− subtype also increased. In comparison, for the ER+/PR+/HER2+ and TN subtypes, as age increased, the percent of cases decreased. The surrogate classification consisted of 71,778 cases of luminal A, 19,011 cases of luminal B/ HER2−, 19,017 cases of luminal B/HER2+, 18,724 cases of triple negative, and 9,792 cases of HER2 overexpressing.

Over 80% of cases were AJCC stages 1 and 2. As stage increased for ER+/PR+/HER2−, the percent of cases decreased. The opposite trend was apparent for all of the HER2+ subtypes except the ER+/PR−/HER2+.

ER+/PR+/HER2− was the majority subtype for all race/ethnicities except for African Americans. Twenty-five percent of blacks had the TN subtype while APIs were more likely to have the ER−/PR−/HER2+ and ER+/PR+/HER2+ subtypes. As SES increased, the percent of cases with ER+/PR+/HER2− increased while the percent of cases decreased for the TN subtype.

A higher percent of cases were tumor grades 1 and 2 as compared to grades 3 and 4. As grade increased, the percent of cases of ER+/PR+/HER2− decreased while ER+/PR+/HER2+, ER−/PR−/HER2+, and TN increased.

The survival analysis and Cox proportional hazards models included AJCC stages 1–3. For this group of patients, 16,340 (8.9%) were missing ER, 21,448 (11.6%) were missing PR, and 45,885 (24.9%) were missing HER2. Approximately 10,700 (6%) of cases were missing grade. SES (3,117), race/ethnicity (1,109), and cause of death (2,726) were missing in less than 2% of cases. Cases were most often missing for patients who were 70 years of age and older, black or Hispanic, and in the lower SES categories. Complete data for all of these variables was available for a total of 123,780 cases: 59,182 in stage 1, 49,982 in stage 2, and 14,616 cases in stage 3.

**Figure 1:**
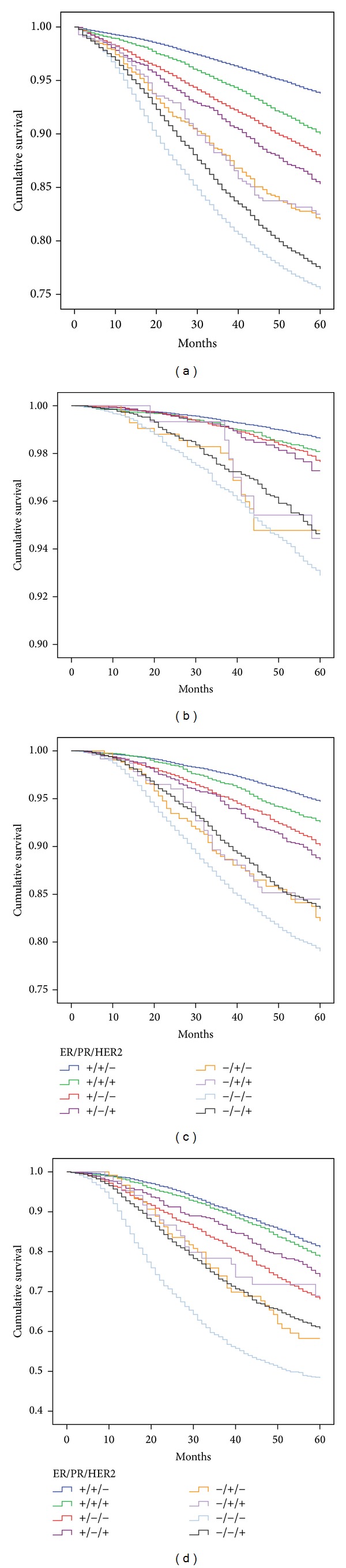
Kaplan Meier 5-year survival for the ER/PR/HER2 subtypes for all stages combined (a), stage 1 (b), stage 2 (c), and stage 3 (d).

**Figure 2:**
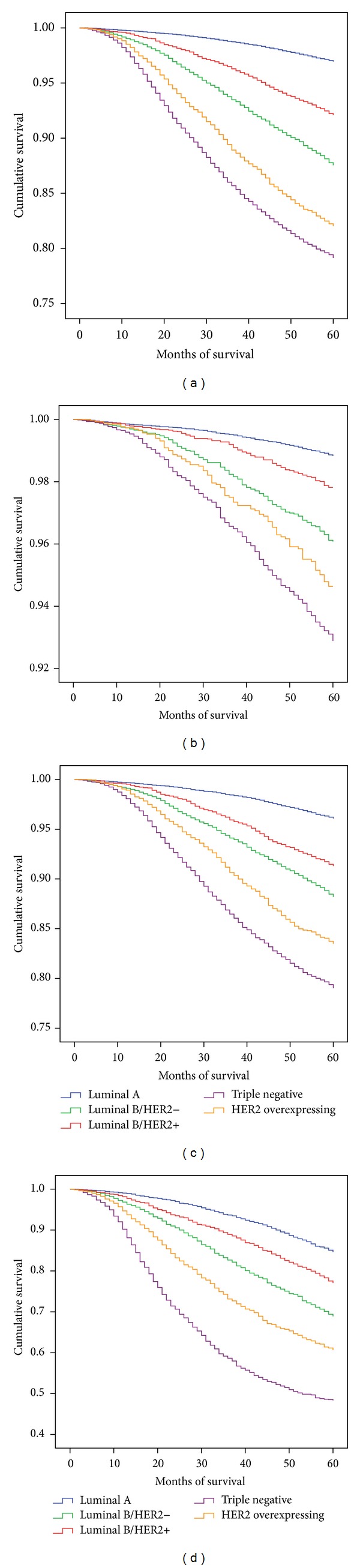
Kaplan Meier 5-year survival for the IHC surrogate classification subtypes for all stages combined (a), stage 1 (b), stage 2 (c), and stage 3 (d).

Results of the survival analysis for the ER/PR/HER2 subtypes and the surrogate classifications are displayed in Figures 1 and 2, respectively. (a) Both figures provide the results for all stages combined, whereas (b) through (d) show the results for stages 1, 2, and 3.

When compared with all stages combined, stratification by stage highlights the clear separation of the ER+ and ER− subtypes. All of the ER+ subtypes had better survival than the ER− subtypes. This difference, while still present in stage 3, was not as dramatic. For stage 1 (b), the worst survival among the ER+ subtypes (ER+/PR−/HER2+) was 97.3% (95%CI = 96.3%, 98.3%), while the ER+/PR+/HER2− had the best survival at 98.6% (95%CI = 98.6%, 98.8%). The TN subtype had the worst survival in all three stages ranging from 92.9% (95%CI = 92.1%, 93.7%) in stage 1 to 48.9% (95%CI = 46.6%, 51.2%) in stage 3 (c). For stages 1 and 2 ((b) and (c)), the ER+/PR+/HER2− subtype had statistically significantly better survival (*P* < 0.001) than all other subtypes. However, the difference between the ER+/PR+/HER2− and ER+/PR+/HER2+ was less than 1% for stage 1 and 2.2% for stage 2. There was no statistically significant difference between the ER+/PR+/HER2− and ER+/PR+/HER2+ subtypes in stage 3.

Additionally, the presence or absence of PR on survival is evident (Figure 1). For example, the survival of the ER+/PR+/HER2− subtype was superior to that of the ER+/PR−/HER2− in all stages. Similarly, the ER+/PR+/HER2+ subtype survival was superior to the ER+/PR−/HER2+ subtype in all stages.

Figure 2 provides the results of the same analysis when the subtypes were defined by the surrogate classification. For all stages (a) and when stratified by stage, luminal A had the best survival when compared with all other subtypes (*P* < 0.001): stage 1 (b), 98.8% (95%CI = 98.7%, 99.0%); stage 2 (c), 96.1% (95%CI = 95.8%, 96.5%); stage 3 (d), 85.0% (95%CI = 83.6%, 86.4%) while the TN subtype had the worst survival in all stages. In stage 1, although statistically significant, there was only a 1% difference in survival between the luminal A (98.8%; 95%CI = 98.7%, 99.0%) and luminal B/HER2+ subtype (97.8%).

**Figure 3:**
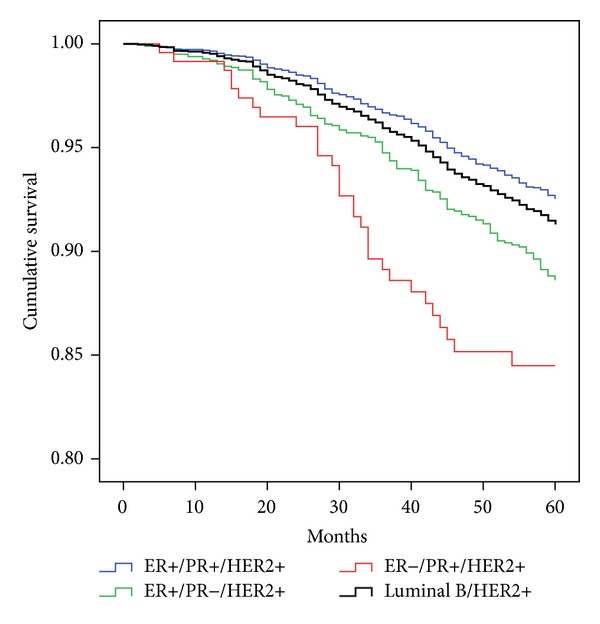
Survival of the luminal B/HER2+ IHC surrogate classification and its ER/PR/HER2 component subtypes for stage 2.

Figure 3 superimposes the survival of the stage 2 luminal B/HER2+ surrogate classification and its component ER/PR/HER2 subtypes (ER+/PR+/HER2+, ER+/PR−/HER2+, and ER−/PR+/HER2+). The luminal B/HER2+ surrogate classification had 91.3% (95%CI = 90.6%, 92.1%) survival which is an approximation of the three component subtypes. Separation of this IHC surrogate demonstrates that there were considerable differences between the three subtypes that comprise this classification. The ER- subtype that makes up this classification had survival that was 8.1% lower than the ER+/PR+/HER2+.

Cox regression analysis for the eight ER/PR/HER2 subtypes demonstrated that with two exceptions, when compared with the ER+/PR+/HER2− subtype, all other subtypes at all stages had an increased risk of mortality (Table 3). The exceptions were in stage 1 where the ER+/PR−HER2− had a similar risk of mortality as the ER+/PR+/HER2−, and in stage 3 where the ER+/PR+HER2+ subtype was not significantly different from the ER+/PR+/HER2−.

Results of the surrogate classification showed that for all stages, all categories had a higher risk of mortality when compared with luminal A, the reference category. In all three stages, the luminal B/HER2− had higher HRs than the luminal B/HER2+. The TN subtype had the highest HRs in all stages.

## 4. Discussion

This study expands on prior research by assessing breast cancer-specific mortality among the ER/PR/HER2 subtypes by stage and contrasts the results with a surrogate classification that incorporates the ER/PR/HER2 subtypes as well as tumor grade. Previous studies indicate that survival of the eight ER/PR/HER2 subtypes is quite variable [10, 30]. The present study demonstrates that this variability is even more striking with stratification by stage. The surrogate classification provides clear separation in survival and adjusted mortality but underestimates the wide variability within the subtypes that make up this classification.

The present study further demonstrates that there is variability in survival among the HER2+ subtypes within each stage of disease [9–11, 13]. While survival was best in all stages for the ER+/PR+HER2− subtype, the difference in survival was less than 1% between ER+/PR+/HER− and ER+/PR+/HER2+ subtypes in stage 1. Differences in tumor biology and clinical outcomes by ER status in HER2+ breast cancer have been shown to exist, and HER2+ breast cancer is acknowledged to be heterogenous adding credence to prior epidemiological findings [10, 31, 32].

The results of this study also demonstrate that when assessing survival using the ER/PR/HER2 subtypes, all of the ER+ subtypes have better survival and adjusted mortality than all of the ER− subtypes with one exception. In stage 3, the ER+/PR−/HER2− subtype has nearly identical survival and adjusted mortality as the ER−/PR+/HER2+ subtype. This suggests that ER may be a more important factor in survival than HER2 and becomes more apparent in stage 3, where the adjusted HR for the ER+/PR+/HER2+ subtype is not different from that of the ER+/PR+/HER2− subtype.

Nuances between the subtypes are not seen when stratifying the subtypes using the surrogate classification. For example, in all stages, the luminal B/HER2− had poorer survival and a higher risk of mortality than the luminal B/HER2+ subtypes. The luminal B/HER2− category consists solely of tumor grades 3 and 4 whereas the luminal B/HER2+ category contains cases of all tumor grades, suggesting that tumor grade, in this instance, may be a more important predictor of mortality than HER2-positivity.

Similarly, the prognostic importance of PR expression can easily be overlooked in the surrogate classification [33]. For example, when one compares the survival of the ER/PR/HER2 subtypes that differ only by the presence or absence of PR, survival of the PR-positive subtype is better in all stages. These important differences are not readily evident when utilizing the surrogate classification.

We recognize that the IHC molecular surrogate classification, currently in vogue, is useful and provides a neat package for reference but does it provide us with more information? For example, luminal B is heterogeneous and relies on either Ki67 or tumor grade. Use of Ki67 has been debated [34–36] and tumor grade is not without its problems, although the genomic grade index may prove to be a valuable alternative [37]. ER/PR/HER2 is universally available and provides excellent breakdown of the subtypes with or without the addition of Ki67 or tumor grade.

We acknowledge the shortcomings/limitations of this population-based cancer registry study [10, 13, 38, 39]. Our research is a retrospective study. The existence of the ER−/PR+/HER2+ and ER−/PR+/HER2− subtypes is controversial [40–45]. However our data, which reflect real-world experience in California, contain 1,711 cases of these two subtypes reported to the CCR. The percent of cases classified as ER−/PR+ has remained constant since 1999 and cannot be ignored [10]. Additionally, these two subtypes had similar survival as the other ER− subtypes. Lastly, we recognize the limitation that Ki67 was unavailable and, although not conclusive, may provide a more precise surrogate classification.

Missing data, especially of HER2, remains a problem. Incomplete or inaccurate race/ethnicity identification, lack of central pathology review, and lack of treatment information are other major limitations. Nonetheless, these types of investigations, especially if conducted with large numbers of patients, may show trends or highlight areas worthy of further research not readily apparent in studies with far fewer patients. We believe our study is of value because of the large number of cases reported to the statewide registry from an ethnically diverse population.

## 5. Conclusions

Gene expression profiling and the latest generation of genomic tests to further explore, decipher, and define the intricacies of tumor biology and categorize the various subtypes of breast cancer are important. However, for the practicing clinician, especially those residing in countries with limited or stressed health care budgets, use of ER, PR, and HER2 is a valuable and acceptable way to subtype breast cancer. Ultimately, it is our hope that classic demographic and clinicopathologic factors be fully integrated with the results of genomic testing. Additionally, linkage of electronic medical records from hospitals/clinics with the CCR would provide medical information sadly lacking at this time [46].

In summary, we have compared the usefulness of the ER/PR/HER2 subtyping of breast cancer with an IHC surrogate classification and, while both are valuable at categorizing breast cancer patients for survival purposes, the ER/PR/HER2 subtype is simple, inexpensive, easy to interpret, reliable, reproducible, and readily available for clinicians without additional tests. Further, the heterogeneity of the HER2+ subtypes and the prognostic importance of ER and PR expression are more readily apparent when using only ER, PR, and HER2.

## Figures and Tables

**Table 1:** Distribution of ER/PR/HER2 and grade that constitute the surrogate molecular classification of combined stages 1–4 breast cancer from the California Cancer Registry 2000–2010.

		Grade 1	Grade 2	Grade 3	Grade 4	Not stated	Total
Luminal A	ER+/PR+/HER2−	23,904 (38.4%)	38,340 (61.6%)				**62,244**
	ER+/PR−/HER2−	3,344 (36.7%)	5,765 (63.3%)				**9,109**
	ER−/PR+/HER2−	117 (27.5%)	308 (72.5%)				**425**

Luminal B, HER2 negative	ER+/PR+/HER2−			13,891 (96.0%)	584 (4.0%)		**14,475**
	ER+/PR−/HER2−			3,694 (95.5%)	175 (4.5%)		**3,869**
	ER−/PR+/HER2−			637 (95.5%)	30 (4.2%)		**667**

Luminal B, HER2 positive	ER+/PR+/HER2+	1,708 (12.4%)	5,803 (42.3%)	5,285 (38.5%)	256 (1.9%)	667 (4.9%)	**13,719**
	ER+/PR−/HER2+	331 (7.0%)	1,789 (37.8%)	2,223 (46.9%)	108 (2.3%)	284 (6.0%)	**4,735**
	ER−/PR+/HER2+	16 (2.8%)	143 (25.4%)	345 (61.3%)	29 (5.2%)	30 (5.3%)	**563**

Triple negative	ER−/PR−/HER2−	508 (2.7%)	3,045 (16.3%)	13,643 (72.9%)	697 (3.7%)	831 (4.4%)	**18,724**

HER2 overexpressing	ER−/PR−/HER2+	159 (1.6%)	2,002 (20.4%)	6,640 (67.8%)	374 (3.8%)	617 (6.3%)	**9,792**

Total		**30,087 (21.8%)**	**57,195 (41.3%)**	**46,358 (33.5%)**	**2,253 (1.6%)**	**2,429 (1.8%)**	**138,322**

**Table 2:** Demographic and tumor characteristics of the ER/PR/HER2 subtypes in women with invasive breast cancer from the California Cancer Registry 2000–2010*^†^.

	ER+/PR+/HER2− 80,765 (57.3%)	ER+/PR+/HER2+ 13,719 (9.6%)	ER+/PR−/HER2− 13,887 (9.7%)	ER+/PR−/HER2+ 4,735 (3.3%)	ER−/PR+/HER2− 1,148 (0.8%)	ER−/PR+/HER2+ 563 (0.4%)	ER−/PR−/HER2− 18,724 (13.1%)	ER−/PR−/HER2+ 9,792 (6.8%)	Total 143,333
Mean age at diagnosis(±SD)	60.78 ± 13.54	56.62 ± 13.86	62.92 ± 13.34	58.99 ± 13.48	54.95 ± 13.76	53.45 ± 13.82	56.80 ± 14.02	56.42 ± 13.33	59.64 ± 13.78

AJCC Stage at diagnosis									
Stage 1	41,004 (64.3%)	5,252 (8.2%)	6,423 (10.1%)	1,634 (2.6%)	466 (0.7%)	172 (0.3%)	6,043 (9.5%)	2,759 (4.3%)	63,753 (45.7%)
Stage 2	28,398 (52.8%)	5,493 (10.2%)	4,842 (9.0%)	1,919 (3.6%)	458 (0.9%)	252 (0.5%)	8,473 (15.8%)	3,911 (7.3%)	53,746 (38.6%)
Stage 3	7,125 (44.2%)	1,825 (11.3%)	1,549 (9.6%)	685 (4.2%)	131 (0.8%)	95 (0.6%)	2,746 (17.0%)	1,982 (12.3%)	16,138 (11.6%)
Stage 4	2,325 (40.7%)	685 (12.0%)	675 (11.8%)	341 (6.0%)	50 (0.9%)	29 (0.5%)	876 (15.3%)	733 (12.8%)	5,714 (4.1%)

Age at diagnosis	*n* (%)	*n* (%)	*n* (%)	*n* (%)	*n* (%)	*n* (%)	*n* (%)	*n* (%)	

<40	4,369 (41.1%)	1,561 (14.7%)	669 (6.3%)	391 (3.7%)	140 (1.3%)	99 (0.9%)	2,305 (21.7%)	1,104 (10.4%)	10,638 (7.4%)
40–49	14,592 (55.4%)	3,238 (12.3%)	1,516 (5.8%)	759 (2.9%)	329 (1.2%)	147 (0.6%)	3,823 (14.5%)	1,940 (7.4%)	26,344 (18.4%)
50–59	20,061 (53.8%)	3,530 (9.5%)	3,583 (9.6%)	1,433 (3.8%)	290 (0.8%)	150 (0.4%)	5,188 (13.9%)	3,068 (8.2%)	37,303 (26.0%)
60–69	19,206 (58.9%)	2,696 (8.3%)	3,633 (11.1%)	1,084 (3.3%)	194 (0.6%)	85 (0.3%)	3,729 (11.4%)	1,997 (6.1%)	32,624 (22.8%)
70–79	14,487 (61.7%)	1,792 (7.6%)	2,816 (12.0%)	681 (2.9%)	126 (0.5%)	55 (0.2%)	2,369 (10.1%)	1,140 (4.9%)	23,466 (16.4%)
80–89	7,239 (62.8%)	800 (6.9%)	1,477 (12.8%)	339 (2.9%)	60 (0.5%)	21 (0.2%)	1,131 (9.8%)	461 (4.0%)	11,528 (8.0%)
90+	811 (56.7%)	102 (7.1%)	193 (13.5%)	48 (3.4%)	9 (0.6%)	6 (0.4%)	179 (12.5%)	82 (5.7%)	1,430 (1.0%)
Race/ethnicity									
White	56,245 (59.5%)	8,455 (8.9%)	9,546 (10.1%)	2,972 (3.1%)	705 (0.7%)	306 (0.3%)	10,801 (11.4%)	5,443 (5.8%)	94,473 (66.3%)
Black	3,557 (41.7%)	811 (9.5%)	859 (10.1%)	302 (3.5%)	88 (1.0%)	48 (0.6%)	2,165 (25.4%)	690 (8.1%)	8,520 (6.0%)
Hispanic	11,693 (50.2%)	2,489 (10.7%)	2,046 (8.8%)	823 (3.5%)	222 (1.0%)	142 (0.6%)	3,830 (16.4%)	2,048 (8.8%)	23,293 (16.3%)
Asian/Pacific Islander	8,496 (54.1%)	1,834 (11.7%)	1,304 (8.3%)	597 (3.8%)	124 (0.8%)	67 (0.4%)	1,767 (11.2%)	1,520 (9.7%)	15,709 (11.0%)
American Indian	268 (52.9%)	55 (10.8%)	49 (9.7%)	23 (4.5%)	4 (0.8%)	0 (0.0%)	74 (14.6%)	34 (6.7%)	507 (0.4%)
Socioeconomic status (SES)									
SES 1—Low	7,990 (49.5%)	1,776 (11.0%)	1,451 (9.0%)	572 (3.5%)	154 (1.0%)	87 (0.5%)	2,676 (16.6%)	1,420 (8.8%)	16,126 (11.4%)
SES 2	12,185 (53.1%)	2,255 (9.8%)	2,192 (9.5%)	802 (3.5%)	179 (0.8%)	99 (0.4%)	3,427 (14.9%)	1,816 (7.9%)	22,955 (16.2%)
SES 3	16,200 (55.9%)	2,763 (9.5%)	2,820 (9.7%)	984 (3.4%)	230 (0.8%)	130 (0.4%)	3,887 (13.4%)	1,991 (6.9%)	29,005 (20.5%)
SES 4	19,392 (57.4%)	3,166 (9.4%)	3,404 (10.1%)	1,098 (3.2%)	254 (0.8%)	117 (0.3%)	4,208 (12.5%)	2,150 (6.4%)	33,789 (23.9%)
SES 5—high	23,792 (60.3%)	3,585 (9.1%)	3,825 (9.7%)	1,219 (3.1%)	317 (0.8%)	126 (0.3%)	4,288 (10.9%)	2,309 (5.9%)	39,461 (27.9%)
Tumor grade									
Well differentiated; Grade I (low)	23,904 (79.4%)	1,708 (5.7%)	3,344 (11.1%)	331 (1.1%)	117 (0.4%)	16 (0.1%)	508 (1.7%)	159 (0.5%)	30,087 (22.1%)
Moderately differentiated; Grade II (low)	38,340 (67.0%)	5,803 (10.1%)	5,765 (10.1%)	1,789 (3.1%)	308 (0.5%)	143 (0.3%)	3,045 (5.3%)	2,002 (3.5%)	57,195 (42.1%)
Poorly differentiated; Grade III (high)	13,891 (30.0%)	5,285 (11.4%)	3,694 (8.0%)	2,223 (4.8%)	637 (1.4%)	345 (0.7%)	13,643 (29.4%)	6,640 (14.3%)	46,358 (34.1%)
Undifferentiated; Grade IV (high)	584 (25.9%)	256 (11.4%)	175 (7.8%)	108 (4.8%)	30 (1.3%)	29 (1.3%)	697 (30.9%)	374 (16.6%)	2,253 (1.7%)
Tumor category									
T1a and microscopic	5,336 (58.7%)	787 (8.7%)	1,025 (11.3%)	337 (3.7%)	68 (0.7%)	42 (0.5%)	691 (7.6%)	806 (8.9%)	9,092 (6.6%)
T1b	15,628 (68.3%)	1,653 (7.2%)	2,402 (10.5%)	516 (2.3%)	146 (0.6%)	46 (0.2%)	1,702 (7.4%)	797 (3.5%)	22,890 (16.6%)
T1c	31,526 (61.7%)	4,801 (9.4%)	4,695 (9.2%)	1,467 (2.9%)	375 (0.7%)	163 (0.3%)	5,615 (11.0%)	2,472 (4.8%)	51,114 (37.1%)
T2	20,782 (49.0%)	4,512 (10.6%)	3,901 (9.2%)	1,620 (3.8%)	392 (0.9%)	204 (0.5%)	7,484 (17.7%)	3,495 (8.2%)	42,390 (30.7%)
T3	5,313 (42.9%)	1,303 (10.5%)	1,268 (10.2%)	539 (4.4%)	106 (0.9%)	59 (0.5%)	2,368 (19.1%)	1,423 (11.5%)	12,379 (9.0%)

*Total cases with ER/PR/HER2; some variables have fewer cases due to missing data.

^†^Percentages are of total cases within a demographic or tumor variable.

**Table 3:** Hazard ratios and 95% confidence intervals derived from Cox regression for ER/PR/HER2 subtype and surrogate classification.

	Stage 1 (*n* = 59,182)	Stage 2 (*n* = 49,982)	Stage 3 (14,616)
ER/PR/HER2*			
ER+/PR+/HER2−	1.00	1.00	1.00
ER+/PR+/HER2+	1.26 (1.04, 1.52)	1.24 (1.12, 1.37)	1.08 (0.94, 1.22)^†^
ER+/PR−/HER2−	1.17 (0.96, 1.43)*	1.50 (1.35, 1.67)	1.66 (1.47, 1.89)
ER+/PR−/HER2+	1.39 (1.03, 1.88)	1.44 (1.25, 1.67)	1.25 (1.04, 1.50)
ER−/PR+/HER2−	1.96 (1.28, 3.02)	1.92 (1.51, 2.43)	1.83 (1.35, 2.48)
ER−/PR+/HER2+	2.21 (1.24, 3.93)	1.75 (1.28, 2.40)	1.65 (1.09, 2.50)
ER−/PR−/HER2−	2.26 (1.93, 2.64)	2.08 (1.92, 2.25)	2.84 (2.58, 3.12)
ER−/PR−/HER2+	1.89 (1.53, 2.31)	1.78 (1.61, 1.97)	1.92 (1.72, 2.34)
Immunohistochemical surrogates for molecular classification**			
Luminal A	1.00	1.00	1.00
Luminal B, HER2−	3.02 (2.58, 3.54)	2.54 (2.33, 2.76)	2.22 (1.99, 2.47)
Luminal B, HER2+	2.02 (1.71, 2.38)	2.06 (1.88, 2.26)	1.66 (1.47, 1.87)
Triple negative	4.68 (4.06, 5.38)	3.93 (3.63, 4.26)	4.45 (4.02, 4.93)
HER2 overexpressing	3.98 (3.29, 4.83)	3.31 (2.99, 3.66)	3.02 (2.70, 3.39)

*Adjusted for age, race/ethnicity, tumor grade, and socioeconomic status.

**Adjusted for age, race/ethnicity, and socioeconomic status.

^†^Confidence intervals that include 1.00 indicate that the risk of mortality within a stage for a subtype was no worse than the reference category.

## Abbreviations

AJCC: American Joint Commission on Cancer
API: Asian/Pacific Islander
CCR: California Cancer Registry
CI: Confidence Interval
ER: Estrogen receptor
HER2: Human epidermal growth factor receptor 2
HR: Hazard ratio
IHC: Immunohistochemical
PR: Progesterone receptor
SES: Socioeconomic status
TN: Triple negative.

## References

[1] Perou CM, Sørile T, Eisen MB (2000). Molecular portraits of human breast tumours. *Nature*.

[2] Sørlie T, Perou CM, Tibshirani R (2001). Gene expression patterns of breast carcinomas distinguish tumor subclasses with clinical implications. *Proceedings of the National Academy of Sciences of the United States of America*.

[3] Carey LA, Perou CM, Livasy CA (2006). Race, breast cancer subtypes, and survival in the Carolina Breast Cancer Study. *Journal of the American Medical Association*.

[4] van de Vijver MJ, He YD, van’t Veer LJ (2002). A gene-expression signature as a predictor of survival in breast cancer. *The New England Journal of Medicine*.

[5] Ma X-J, Wang Z, Ryan PD (2004). A two-gene expression ratio predicts clinical outcome in breast cancer patients treated with tamoxifen. *Cancer Cell*.

[6] Paik S, Shak S, Tang G (2004). A multigene assay to predict recurrence of tamoxifen-treated, node-negative breast cancer. *The New England Journal of Medicine*.

[7] Sotiriou C, Wirapati P, Loi S (2006). Gene expression profiling in breast cancer: understanding the molecular basis of histologic grade to improve prognosis. *Journal of the National Cancer Institute*.

[8] Badve SS, Baehner FL, Gray RP (2008). Estrogen- and progesterone-receptor status in ECOG 2197: comparison of immunohistochemistry by local and central laboratories and quantitative reverse transcription polymerase chain reaction by central laboratory. *Journal of Clinical Oncology*.

[9] Brown M, Tsodikov A, Bauer KR, Parise CA, Caggiano V (2008). The role of human epidermal growth factor receptor 2 in the survival of women with estrogen and progesterone receptor-negative, invasive breast cancer: The California Cancer Registry, 1999–2004. *Cancer*.

[10] Parise CA, Bauer KR, Brown MM, Caggiano V (2009). Breast cancer subtypes as defined by the estrogen receptor (ER), progesterone receptor (PR), and the human epidermal growth factor receptor 2 (HER2) among women with invasive breast cancer in California, 1999–2004. *The Breast Journal*.

[11] Bauer K, Parise C, Caggiano V (2010). Use of ER/PR/HER2 subtypes in conjunction with the 2007 St Gallen consensus statement for early breast cancer. *BMC Cancer*.

[12] Collins LC, Schnitt SJ, Colditz GA (2006). Risk factors for luminal, HER2, and basal-like breast cancer subtypes: results from a tissue microarray-based analysis of invasive breast cancers from women enrolled in the nurses' health study. *Breast Cancer Research and Treatment*.

[13] Bauer KR, Brown M, Cress RD, Parise CA, Caggiano V (2007). Descriptive analysis of estrogen receptor (ER)-negative, progesterone receptor (PR)-negative, and HER2-negative invasive breast cancer, the so-called triple-negative phenotype: a population-based study from the California Cancer Registry. *Cancer*.

[14] Linn SC, Van ’t Veer LJ (2009). Clinical relevance of the triple-negative breast cancer concept: genetic basis and clinical utility of the concept. *European Journal of Cancer*.

[15] Tamimi RM, Baer HJ, Marotti J (2008). Comparison of molecular phenotypes of ductal carcinoma in situ and invasive breast cancer. *Breast Cancer Research*.

[16] Bhargava R, Dabbs DJ (2008). Luminal B breast tumors are not HER2 positive. *Breast Cancer Research*.

[17] Cheang MCU, Chia SK, Voduc D (2009). Ki67 index, HER2 status, and prognosis of patients with luminal B breast cancer. *Journal of the National Cancer Institute*.

[18] Goldhirsch A, Wood WC, Coates AS, Gelber RD, Thürlimann B, Senn H-J (2011). Strategies for subtypes-dealing with the diversity of breast cancer: highlights of the St Gallen international expert consensus on the primary therapy of early breast cancer 2011. *Annals of Oncology*.

[19] Brouckaert O, Schoneveld A, Truyers C (2013). Breast cancer phenotype, nodal status and palpability may be useful in the detection of overdiagnosed screening-detected breast cancers. *Annals of Oncology*.

[20] Fritz AG (2000). *International Classification of Diseases for Oncology: ICD-O-3*.

[21] (2008). *Cancer Reporting in California: Abstracting and Coding Procedures for Hospitals. CaliFornia Cancer Reporting System Standards*.

[22] Yost K, Perkins C, Cohen R, Morris C, Wright W (2001). Socioeconomic status and breast cancer incidence in California for different race/ethnic groups. *Cancer Causes and Control*.

[23] Clarke CA, Glaser SL, Keegan THM, Stroup A (2005). Neighborhood socioeconomic status and Hodgkin’s lymphoma incidence in California. *Cancer Epidemiology Biomarkers and Prevention*.

[24] Parikh-Patel A, Bates JH, Campleman S (2006). Colorectal cancer stage at diagnosis by socioeconomic and urban/rural status in California, 1988–2000. *Cancer*.

[25] Zell JA, Rhee JM, Ziogas A, Lipkin SM, Anton-Culver H (2007). Race, socioeconomic status, treatment, and survival time among pancreatic cancer cases in California. *Cancer Epidemiology Biomarkers and Prevention*.

[26] Ignatius Ou S-H, Zell JA, Ziogas A, Anton-Culver H (2008). Low socioeconomic status is a poor prognostic factor for survival in stage I nonsmall cell lung cancer and is independent of surgical treatment, race, and marital status. *Cancer*.

[27] Yin D, Morris C, Allen M, Cress R, Bates J, Liu L (2010). Does socioeconomic disparity in cancer incidence vary across racial/ethnic groups?. *Cancer Causes and Control*.

[28] Parise CA, Caggiano V (2013). Disparities in race/ethnicity and socioeconomic status: risk of mortality of breast cancer patients in the California Cancer Registry, 2000–2010. *BMC Cancer*.

[29] Parise CA, Bauer KR, Caggiano V (2012). Disparities in receipt of adjuvant radiation therapy after breast-conserving surgery among the cancer-reporting regions of California. *Cancer*.

[30] Parise CA, Bauer KR, Caggiano V (2010). Variation in breast cancer subtypes with age and race/ethnicity. *Critical Reviews in Oncology/Hematology*.

[31] Vaz-Luis I, Winer EP, Lin NU (2013). Human epidermal growth factor receptor-2-positive breast cancer: does estrogen receptor status define two distinct subtypes?. *Annals of Oncology*.

[32] Montemurro F, Di Cosimo S, Arpino G (2013). Human epidermal growth factor receptor 2 (HER2)-positive and hormone receptor-positive breast cancer: new insights into molecular interactions and clinical implications. *Annals of Oncology*.

[33] Purdie CA, Quinlan P, Jordan LB (2014). Progesterone receptor expression is an independent prognostic variable in early breast cancer: a population-based study. *British Journal of Cancer*.

[34] Colozza M, Sidoni A, Piccart-Gebhart M (2010). Value of Ki67 in breast cancer: the debate is still open. *The Lancet Oncology*.

[35] Ferguson NL, Bell J, Heidel R (2013). Prognostic value of breast cancer subtypes, Ki-67 proliferation index, age, and pathologic tumor characteristics on breast cancer survival in Caucasian women. *The Breast Journal*.

[36] Lips EH, Mulder L, de Ronde JJ (2013). Breast cancer subtyping by immunohistochemistry and histological grade outperforms breast cancer intrinsic subtypes in predicting neoadjuvant chemotherapy response. *Breast Cancer Research and Treatment*.

[37] Metzger-Filho O, Catteau A, Michiels S (2013). Genomic Grade Index (GGI): feasibility in routine practice and impact on treatment decisions in early breast cancer. *PloS ONE*.

[38] Izquierdo JN, Schoenbach VJ (2000). The potential and limitations of data from population-based state cancer registries. *American Journal of Public Health*.

[39] Edwards BK, Brown ML, Wingo PA (2005). Annual report to the nation on the status of cancer, 1975–2002, featuring population-based trends in cancer treatment. *Journal of the National Cancer Institute*.

[40] Rakha EA, El-Sayed ME, Green AR (2007). Biologic and clinical characteristics of breast cancer with single hormone receptor-positive phenotype. *Journal of Clinical Oncology*.

[41] De Maeyer L, van Limbergen E, de Nys K (2008). Does estrogen receptor-negative/progesterone receptor-positive breast carcinoma exist?. *Journal of Clinical Oncology*.

[42] Cserni G, Francz M, Kálmán E (2011). Estrogen receptor negative and progesterone receptor positive breast carcinomas-how frequent are they?. *Pathology and Oncology Research*.

[43] Ng CH, Pathy NB, Taib NA, Mun KS, Rhodes A, Yip CH (2012). The estrogen receptor negative-progesterone receptor positive breast carcinoma is a biological entity and not a technical artifact. *Asian Pacific Journal of Cancer Prevention*.

[44] Hefti MM, Hu R, Knoblauch NW (2013). Estrogen receptor negative/progesterone receptor positive breast cancer is not a reproducible subtype. *Breast Cancer Research*.

[45] Iwamoto T, Matsuoka J, Nogami T Estrogen receptor (ER) mRNA expression and molecular subtype distribution in breast cancers that are ER-negative but progesterone receptor-positive by immunohistochemistry.

[46] Kurian AW, Mitani A, Desai M (2014). Breast cancer treatment across health care systems: linking electronic medical records and state registry data to enable outcomes research. *Cancer*.

